# Effects of Drought, Heat and Their Interaction on the Growth, Yield and Photosynthetic Function of Lentil (*Lens culinaris* Medikus) Genotypes Varying in Heat and Drought Sensitivity

**DOI:** 10.3389/fpls.2017.01776

**Published:** 2017-10-17

**Authors:** Akanksha Sehgal, Kumari Sita, Jitendra Kumar, Shiv Kumar, Sarvjeet Singh, Kadambot H. M. Siddique, Harsh Nayyar

**Affiliations:** ^1^Department of Botany, Panjab University, Chandigarh, India; ^2^Indian Institute of Pulses Research, Kanpur, India; ^3^International Center for Agricultural Research in the Dry Areas, Rabat, Morocco; ^4^Plant Breeding and Genetics, Punjab Agricultural University, Ludhiana, India; ^5^The UWA Institute of Agriculture, The University of Western Australia, Perth, WA, Australia

**Keywords:** water stress, high temperature, photosynthesis, reproductive growth, sucrose, starch, carbohydrates

## Abstract

Rising temperatures and drought stress limit the growth and production potential of lentil (*Lens culinaris* Medikus), particularly during reproductive growth and seed filling. The present study aimed to (i) investigate the individual and combined effects of heat and drought stress during seed filling, (ii) determine the response of lentil genotypes with contrasting heat and drought sensitivity, and (iii) assess any cross tolerance in contrasting genotypes. For this purpose, eight lentil genotypes (two drought-tolerant, two drought-sensitive, two heat-tolerant, two heat-sensitive) were either sown at the normal time (second week of November 2014), when the temperatures at the time of seed filling were below 30/20°C (day/night), or sown late (second week of February 2015) to impose heat stress (temperatures > 30/20°C (day/night) during reproducive growth and seed filling. Half of the pots in each sowing environment were fully watered throughout (100% field capacity) while the others had water withheld (50% of field capacity) from the start of seed filling to maturity. Both heat and drought, individually or in combination, damaged cell membranes, photosynthetic traits and water relations; the effects were more severe with the combined stress. RuBisCo and stomatal conductance increased with heat stress but decreased with drought and the combined stress. Leaf and seed sucrose decreased with each stress in conjunction with its biosynthetic enzyme, while its (sucrose) hydrolysis increased under heat and drought stress, but was inhibited due to combination of stresses. Starch increased under heat stress in leaves but decreased in seeds, but drastically declined in seeds under drought alone or in combination with heat stress. At the same time, starch hydrolysis in leaves and seeds increased resulting in an accumulation of reducing sugars. Heat stress inhibited yield traits (seed number and seed weight per plant) more than drought stress, while drought stress reduced individual seed weights more than heat stress. The combined stress severely inhibited yield traits with less effect on the drought- and heat-tolerant genotypes. Drought stress inhibited the biochemical processes of seed filling more than heat stress, and the combined stress had a highly detrimental effect. A partial cross tolerance was noticed in drought and heat-tolerant lentil genotypes against the two stresses.

## Introduction

High temperatures and water deficit conditions are major environmental factors, which frequently limit the growth and productivity of important crop species (Barnabás et al., 2008). The effects of heat and drought stress on crops is well documented; however, experiments that combine heat and drought stress are not common despite the fact that heat and drought are strongly coupled and have deleterious effects on crop growth and productivity (Cvikrová et al., 2013; Lipiec et al., 2013; Rollins et al., 2013; Shinohara and Leskovar, 2014; Lobell et al., 2015; Nankishore and Farrell, 2016). Global climate change is an added concern, increasing overall temperatures, altering the distribution of precipitation and aggravating drought conditions in semiarid and arid areas (Turner and Meyer, 2011; Awasthi et al., 2014) and, ultimately, compromising crop productivity in numerous regions worldwide (Bai et al., 2004; Müller et al., 2009; Awasthi et al., 2014). The prevalence of drought, accompanied by high temperatures, is expected to increase in the near future (IPCC, 2014), and emphasizes the need to study this stress combination to enhance the tolerance in future crops (Zandalinas et al., 2017). The effects of heat and drought stress in combination have been studied in some crops, e.g., canola (*Brassica napus* L.), groundnut (*Arachis hypogea* L.) (Hamidou et al., 2013), wheat (*Triticum aestivum* L.) (Wardlaw, 2002), maize (*Zea mays* L.) (Cairns et al., 2013) and chickpea (*Cicer arietinum* L.) (Canci and Toker, 2009; Awasthi et al., 2014), but not in lentil (*Lens culinaris* Medikus).

The combined effects of drought and heat on plant growth and productivity are more severe than those of the individual effects (Barnabás et al., 2008; De Boeck et al., 2015; Zandalinas et al., 2016a,b) and the reproductive stages are more susceptible to drought, heat and the combined stress than the vegetative stages (Barnabás et al., 2008). In cereals such as wheat and maize, drought and heat stress reduced photosynthesis, stomatal conductance, leaf area and water-use efficiency (Shah and Paulsen, 2003). These stresses appearing at the time of flowering and anthesis result in fertilization failures due to reduced pollen and ovule function and inhibited pollen development and sterility (Prasad et al., 2008). Combined heat and drought stress adversely affect the reproductive processes in cereals (Barnabás et al., 2008; Prasad et al., 2011) and legumes such as groundnut (Prasad et al., 2000; Sadras et al., 2013) and chickpea (Awasthi et al., 2014). Little information is available on the physiological and biochemical responses of food legumes to combined heat and drought stress, which need further investigation to understand the mechanisms of stress tolerance.

Seed development is a crucial growth stage in all grain crops as it engages processes to import contents from leaves, and associated biochemical processes required for the synthesis of various macromolecules (carbohydrates, proteins and lipids) in seeds (Ahmadi and Baker, 2001; Behboudian et al., 2001; Triboï et al., 2003). Drought accompanied by heat stress during seed development and filling reduces yield, as observed in legumes (Canci and Toker, 2009; Awasthi et al., 2014) and cereals (Barnabás et al., 2008). During seed filling, sucrose metabolism is crucial in leaves and seeds, as it plays an important role in the hexose–sucrose balance that regulates essential aspects of seed development (Weschke et al., 2000). In maize, the activities of vacuolar and cell-wall-bound acid invertases dominate during kernel development (Weschke et al., 2000), which decreased during drought stress (Zinselmeier et al., 1999; Andersen et al., 2002). Further, drought can impair seed filling due to the disruption of metabolic pools downward of sucrose in the starch synthesis (Zinselmeier et al., 1999). Thus, the combination of heat and drought stress may further influence the transfer of assimilates needed for seed filling.

Lentil (*Lens culinaris* Medik.) is a major cool season food legume in India and the second most important winter-season legume after chickpea (*Cicer arietinum* L.) (Kumar et al., 2016). It requires low temperatures during vegetative growth, while at maturity, warm temperatures are required; the ‘optimum temperature for its best growth has been reported to be 18–30°C’ (Sinsawat et al., 2004; Roy et al., 2012). Of the abiotic stresses experienced by lentil worldwide, drought and heat stress are considered the most important (Singh and Saxena, 1993). The susceptibility of lentil to hot and semiarid regions is supported by many researchers (Erskine and El Ashkar, 1993; Oktem et al., 2008; Barghi et al., 2012; Allahmoradi et al., 2013). In India, most of the lentil sowings get postponed because of the delayed harvest of the preceding crop, which generally happens to be paddy, especially in northern part of India. As a result, the crop at the time of seed-filling stage suffers due to the rising high temperatures in most of its cultivated areas. In Indo-Gangetic region, Rajasthan, Maharashtra (India) etc. lentil is grown comparatively at higher temperatures. During the seed-filling stage, the crop is usually adversely affected by the high approaching summer temperatures, leading to low grain yields and poor grain quality (Tickoo et al., 2005).

Heat stress is also accompanied by drought stress due to rapid water loss from the soil and plants (Wahid et al., 2007). Consequently, lentil may face the combined effects of heat and drought stress, especially during seed filling, which can adversely impact its yield components. No information exists on this aspect in lentil, thus, the present study was undertaken to: (a) investigate the effects of heat and drought stress, individually and in combination, on the biochemical processes related to seed filling and yield components, (b) identify variation in processes related to seed filling in genotypes varying in heat and drought sensitivity, and (iii) find out any cross tolerance in contrasting lentil genotypes for these two stresses.

## Materials and Methods

### Genotypes

Eight contrasting lentil genotypes—two drought-tolerant (DT; DPL53 and JL1), two drought-sensitive (DS; ILL 2150 and ILL 4345), two heat-tolerant (HT; 1G 2507 and 1G 4258) and two heat-sensitive (HS; 1G 3973 and 1G 3964)—with matching phenology were sourced from ICARDA Morocco and the Indian Institute of Pulse Research, Kanpur, India, and grown in earthen pots. The details of these genotypes are shown in **Table 1**.

**Table 1:** Details on the source and yield of lentil genotypes used in the study.

Genotypes	100-seed weight	Source
DPL 53 (DT)	2.5	India
JL1 (DT)	2.3	India
ILL 2150 (DS)	2.3	Jordan
ILL 4345 (DS)	1.5	India
1G 2507 (HT)	2.0	India
1G 4258 (HT)	2.0	India
1G 3973 (HS)	1.6	India
1G 3964 (HS)	1.9	India

*DT is drought tolerant, DS is drought sensitive, HT is heat tolerant, HS is heat sensitive.*

### Planting Conditions

Lentil plants were grown in pots in natural outdoor conditions. There were two sowing dates—the second week of November (12th November 2014 for normal sowing (NS) and the second week of February (10th February 2015) for late sowing (LS). The late-sowing treatment ensured heat stress (>30/20°C; average max/min temperatures) during seed filling. The plants were grown outdoors in a wire-covered dome to minimize bird and animal damage at Panjab University, Chandigarh, India (30°44′5.9994″ N, 76°47′27.5994″ E). The eight lentil genotypes were sown in earthen pots (8 kg soil capacity; 300 mm diameter). The pots were filled with a mixture of sandy loam soil and sand in a 3:1 ratio with one part manure and three parts of the soil–sand mixture along with 10 mg kg^-1^ tricalcium phosphate fertilizer. Seeds were inoculated with *Rhizobium* spp. before sowing. Ten seeds were sown in each pot and thinned to five per pot 20 days after sowing (DAS). There were 5 pots per genotype, with three replications for each of the four treatments. The pots were completely randomized. Daily maximum and minimum temperatures were recorded for the duration of the experiment. Relative humidity, vapor pressure deficit (VPD) and photoperiod were also recorded.

**FIGURE 1:**
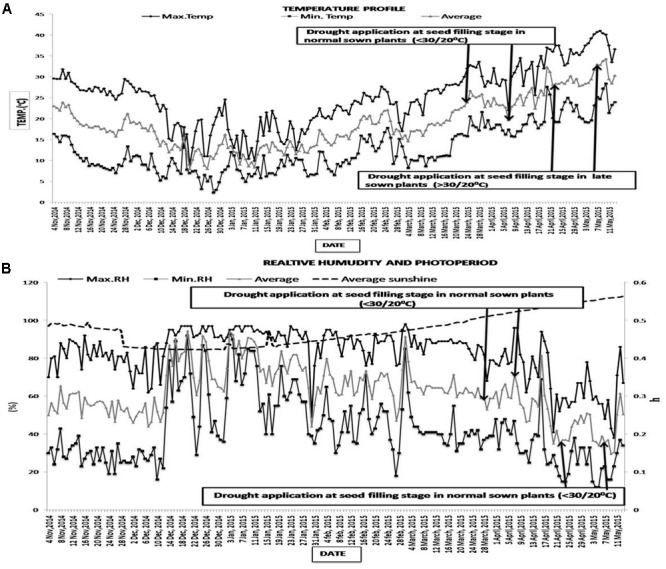
**(A)** Maximum, minimum and mean air temperatures (°C), and **(B)** relative humidity (%), photoperiod (h) at the experimental site from 4 November 2014 to 13 May 2015. The arrows show the time that the drought treatment was imposed (first arrow) and completed (second arrow) on lentil sown in November 2014 (normal sowing) and February 2015 (late sowing).

The maximum and minimum temperatures during seed filling for the NS treatment were below 30/20°C [22–30°C (day)/16–19°C (night)] while those of the LS treatment were above 30/20°C [32–40°C (day)/21–27°C (night)]. The photoperiod varied from 12.1–12.5 h during the normal-sown (NS) growing season and 13.0–13.3 h during the late-sown growing season. Relative humidity ranged from 95–32% in NS plants and 78–12% in LS plants (**Figure 1**). VPD ranged from 2.3–3.1 kPa in NS plants to 3.3–5.1 kPa in LS plants

### Drought and Heat Stress Treatments

The plants were irrigated daily (∼100% field capacity) to prevent water deficit up to seed filling When the plants were at 75% podding (∼120 DAS in NS and 65 DAS in late-sown plants), drought stress was imposed by reducing soil moisture to 50% field capacity until maturity (15 days in both sowing environments) (Awasthi et al., 2014). Soil moisture was checked daily with soil moisture probe (Field Scout TDR 300 Probe, Spectrum Technologies, Inc., United States), and gravimeteric method periodically to maintain 50% field capacity.

Thus, there were the following four treatments:

(1) Unstressed control (NS plants, fully irrigated to ∼100% field capacity)
(2) Drought stress (NS plants, fully irrigated until 75% podding then maintained at ∼50% field capacity)
(3) Heat stress (LS plants, fully irrigated to ∼100% field capacity)
(4) Drought and heat stress (LS plants, fully irrigated until 75% podding then maintained at ∼50% field capacity)

### Sample Collection

For analyses of biomass and yield parameters, three plants per pot were harvested at maturity. Aboveground biomass, yield and yield constituents such as pod number per plant, seed number per plant and mean individual seed weight were recorded. Plants were cut at soil level, and the number of seeds and filled pods were counted before being oven-dried for 3 days at 45°C, and weighed. The average values of the three plants per pot were presented on a per-plant basis.

For stress injury and biochemical parameters, seeds and leaves (preferably from the second and third branches from the top) were collected randomly from three plants per genotype × treatment combination at 11:00 am at the end of stress period.

Samples from the late-sown plants were collected after the plants had experienced elevated temperatures (as described above in planting conditions) along with water stress for a minimum of 15 consecutive days. The plants used for biochemical tests were not included for measuring yield traits. The samples for analysis of leaves and seeds were collected at the end of stress period (15 days) in both sowing environments.

### Stress Injury Analysis

Stress injury to leaves was estimated as electrolyte leakage (Premchandra et al., 1990) to measure the permeability of the cell membrane (Lutts et al., 1996; Sita et al., 2017). Fresh samples of leaves (100 mg) were washed thrice with deionized water, placed in closed vials containing 10 mL deionized water, and incubated at 25°C overnight. The electrical conductivity of the bathing solution was measured 24 h later with a conductivity meter (ELICO CM 180, Hyderabad, India) and expressed in mmhos g^-1^ dry weight (DW).

### Photosynthetic Components

Leaf photosynthesis was measured at the end of the stress period on intact leaves from the second and third branches from the top using an infrared gas analyzer (Qubit Systems, Canada) at 11:00 h. ‘PSII activity on leaves from the same branches was evaluated as chlorophyll fluorescence using the dark-adapted test of the modulated chlorophyll fluorometer (OS1-FL, Opti-Sciences, Tyngsboro, MA, United States) at 11:00 h. With this system, chlorophyll fluorescence is excited by a 660-nm solid-state light source, with filters blocking radiation at wavelengths above 690 nm. The average intensity of this modulated light was adjusted from 0 to 1 μE. The observation was done in a range of 700–750 nm with the help of PIN silicon photodiode with appropriate filtering to avoid external light. The clamps of the instrument were positioned on the leaves to keep them in the dark and to halt the light reaction of photosynthesis for 45 min. The clamps were then attached to the optic fiber of the device and the valves were opened. The device was turned on, and the 695 nm modulated light was radiated through the optic fiber toward the leaf. The Fv/Fm ratio (the maximum quantum yield of PSII photochemistry) was recorded as an expression of PSII activity’ (Awasthi et al., 2014).

Chlorophyll concentration was tested from the same leaves which were used for measuring Pn and PS II function. ‘The fresh leaves (100 mg) were homogenized in 80% acetone, and centrifuged at 5,701.8 *g* for 10 min. The absorbance of the supernatant was read at 645 and 663 nm. Total chlorophyll was measured against 80% acetone as blank’ (Arnon, 1949; Awasthi et al., 2014). Chlorophyll was extracted from fresh leaves but was expressed on DW basis to rule out any changes due to altered water status. For measuring DW, a separate lot of fresh leaves having fresh weight similar to the leaves used for chlorophyll extraction were dried in hot air oven hot air over at 45°C for 2 days.

The chlorophyll concentration (mmol g^-1^ DW) was measured using the following three equations:

$$\begin{array}{c} \mathrm{Chl}\;a\;=\;12.9\;({\mathrm{Abs}}_{663})\;-2.69\;({\mathrm{Abs}}_{645})\;\frac{V}{1000\times W}\\ \mathrm{Chl}\;b\;=\;22.9\;({\mathrm{Abs}}_{645})\;-4.68\;({\mathrm{Abs}}_{663})\;\frac{V}{1000\times W}\\ \mathrm{Total}\;\mathrm{Chl}\;=\;\mathrm{Chl}\;a\;+\;\mathrm{Chl}\;b \end{array}$$

where V = final volume (ml), W = tissue DW (g), Abs_663_ = absorbance at 663 nm and Abs_645_ = absorbance at 645 nm.

For measuring chlorophyll content and other biochemical parameters, fresh samples were collected, but the calculations were made on DW basis. Fresh material was oven-dried at 45°C for 2 days, and its DW was measured.

### Water Relations

Relative leaf water content (RLWC) was calculated according to the method of Barrs and Weatherley (1962). Leaf samples (4–5 leaves from top 2 or 3 branches) from each genotype were collected, weighed (fresh weight), immersed in distilled water for 3 h in a Petri dish, removed and weighed (turgid weight), and then oven-dried at 80°C for 24 h and reweighed (DW).

RLWC was calculated using formula:

$$\begin{array}{c} \mathrm{RLWC}\;(\%)\;=\;\frac{\mathrm{Freshwt}\;-Drywt.}{\mathrm{Turgidwt}\;-Drywt.}\;\times \;100 \end{array}$$

Osmotic potential of the leaves was measured using an osmometer (Wescor, United States). ‘The stomatal conductance (gs) of fully expanded leaves (from the second or third branches from the top) was measured using a portable leaf porometer (model SC1, Decagon Devices, Pullman, WA, United States) at 11:00 h at the end of the stress period and expressed as mmol m^-2^ s^-1^′ (Awasthi et al., 2014).

### Enzyme Analysis

The photosynthetic function of leaves (from the second and third branches from the top) was measured on the basis of activities of a photosynthetic enzyme (RuBisCo), a sucrose-synthesizing enzyme (sucrose phosphate synthase), a sucrose catabolic enzyme (vacuolar acid invertase), a starch-synthesizing enzyme (starch phosphorylase) and a starch-hydrolyzing enzyme (β-amylase), and sucrose concentration.

‘To estimate the activity of RuBisCo, fresh leaves were homogenized in a pre-cooled mortar and pestle in a buffer solution comprising 50 mM 1,3-bis tris (hydroxymethyl) methylamino propane (pH = 7.0), 10 nM NaHCO_3,_ 10 mM MgCl_2_, 1 mM EDTA, 0.5 mM ATP, 10 mM DTT, 1 mM phenylmethyl-sulfonyl fluoride, 1 mM benzamidine, 1.5% polyvinyl polypyrrolidone and 3 mM 3-methylbut-2-ene-1-thiol, according to the method of Wang et al. (1992). The leaf extract was centrifuged at 29,068 *g* for 40 min. The supernatant was de-salted immediately at 4°C by passing it through 4 mL Sephadex G-25 columns (Sigma, St. Louis, MO, United States) pre-equilibrated with buffer solution containing 20 mM HEPES–NaOH (pH 7.5), 0.25 mM MgCl_2_, 0.01% 2-mercaptoethanol, 1 mM EDTA and 0.05% BSA. The de-salted extract was assayed immediately using the method of Racker (1962). The assay medium contained 1 M Tris buffer (pH 7.8), 0.006 M NADH, 0.1 M reduced glutathione, 0.5% glyceraldehyde-3- phosphate dehydrogenase, 0.025 M 3-phosphoglycerate kinase, 0.05% a-glycerophosphate dehydrogenase-triose phosphate isomerase, 0.025 M ribulose 1–5 biphosphate, 0.2 M ATP, 0.5 M MgCl_2_ and 0.5 M KHCO_3_. The enzyme extract was added to the assay medium to make a final volume of 1 mL. The oxidation of NADH was observed at 340 nm during the conversion of 3-phosphoglycerate to glycerol 3-phosphate using a molar extinction coefficient of 6.22 mM cm^-1^. One unit was taken as the amount that catalyzed the cleavage of 1 mM RuBP per min. The reaction was monitored for 3 min at 25°C until there was a uniform change in the absorbance. RuBisCo activity was expressed as mmol NADH oxidized g^-1^ DW min^-1^′ (Awasthi et al., 2014).

To assay the enzymes related to sucrose and starch metabolism, leaf and seed tissues collected from the control and stressed plants at the end of the stress period. ‘These were homogenized in a ice-cold HEPES buffer solution containing 50 mM L^-1^ NaOH (pH 7), 2 mM L^-1^ MgCl_2_, 1 mM L^-1^ EDTA and 2 mM L^-1^ DTT’ (Dejardin et al. (1997). The homogenate was centrifuged at 16,350 *g* in a cold centrifuge for 20 min. The supernatant was de-salted quickly at 4°C by passing it through 4 mL Sephadex G-25 columns pre-equilibrated with a buffer solution containing 20 mM HEPES–NaOH (pH 7.5), 0.25 mM MgCl_2_, 0.01% 2-mercaptoethanol, 1 mM EDTA and 0.05% BSA. The de-salted extract was assayed immediately.

β-amylase activity was measured using the method of Shuster and Gifford (1962). ‘The reaction mixture containing 0.2 mL enzyme extract and 1 mL freshly prepared starch solution (0.2%) was incubated for 1 h at 30°C. The reaction was ended by the addition of 1 mL of 3,5-dinitrosalicylic acid (DNSA) reagent. The tubes were then boiled for 10 min and cooled to room temperature before adding 2 mL of distilled water to each test tube. The absorbance at 560 nm was measured using glucose as a standard. To check level of endogenous sugars, a control was run for each reaction mixture. The activity was recorded for a standard curve of glucose and expressed as mmol glucose formed g^-1^ DW’ (Awasthi et al., 2014).

Starch phosphorylase activity was assayed in both leaves and seeds according to the method of Baun et al. (1970). ‘To 0.2 mL of enzyme extract, 0.6 mL Tris-maleate buffer (pH 6.5) containing 1 mM NaF was added followed by 0.2 mL of 0.05 M glucose-1-phosphate. The reaction mixture was then incubated at 30°C for 1 h. The reaction mixture was ended by the addition of 0.5 mL chilled 5% trichloroacetic acid. The mixture was centrifuged at 29,068 *g* to settle protein precipitate. To calculate the inorganic phosphate, 3.3 mL distilled water and 1 mL ammonium molybdate reagent (1.5 g ammonium molybdate + 30 mL conc. HCl, diluted to 100 mL with distilled water) were added to 0.5 mL of supernatant. The test-tubes were shaken well, and after ∼5 min, 0.2 mL of Fiske and Subbarow reagent (1.45 g sodium metabisulfite + 50 mg sodium sulfite + 25 mg 1-amino-2-napthol-2-sulfonic acid dissolved in 5 mL water to make a final volume of 10 mL) was added. Simultaneously, blanks were run with the heat-inactivated enzyme extract. The mixture was then incubated at 30°C for 15 min, and absorbance measured at 660 nm using monopotassium phosphate as a standard. The activity was assayed from the standard curve of mono potassium phosphate and expressed as nmol inorganic phosphate min^-1^ g^-1^ DW’ (Awasthi et al., 2014).

Sucrose synthase activity was assayed as described by Hawker et al. (1976). ‘To the reaction mixture, (0.015 M uridine diphosphate glucose + 0.05 M fructose + 0.2 M tris-HCl buffer (pH 8.2), enzyme extract was added. The above mixture was incubated for 30 min at 37°C; the reaction was terminated by heating the tubes in a boiling water bath for 10 min and cooled to room temperature. Residual fructose was destroyed by adding 0.5 mL of 6% KOH. The contents were then heated in a boiling water bath for 20 min and cooled to room temperature. One mL of 1% resorcinol solution and 3 mL of 30% HCl were added to the tubes which were then incubated for 10 min at 80°C. The intensity of the developed pink color was observed at 490 nm. Simultaneously, the blanks were run with heat-inactivated enzyme extract. Sucrose concentration was calculated from the standard curve of sucrose (40–280 mg mL^-1^) and expressed as mmol sucrose g^-1^ DW h^-1^′ (Awasthi et al., 2014).

Vacuolar acid invertase activity was measured using the method of Nygaard (1977). ‘Enzyme extract (0.1 ml) was added to the reaction mixture comprising 0.6 mL of 0.2 M acetate buffer (pH 4.8) and 0.3 mL of 0.4 M sucrose solution (prepared in 0.2 M acetate buffer). Sucrose in the control tubes was added only after the enzyme preparation had been inactivated by boiling for 5 min. After incubating for 30 min at 30°C, to the reaction mixture 1 mL DNSA was added. The tubes were then placed for 10 min in a boiling water bath and cooled down to room temperature. The samples were diluted to 5 mL with distilled water and absorbance measured at 560 nm using glucose as a standard. Simultaneously, blanks were run with the heat-inactivated enzyme extract. The activity was calculated from the standard curve of glucose and expressed as mmol glucose g^-1^ DW h^-1^)’ (Awasthi et al., 2014).

### Sucrose, Starch and Reducing Sugars

Sucrose concentration was assayed as per the enzymatic method of Jones et al. (1977). ‘Fresh leaf and seed samples were extracted in 80% ethanol three times at 80°C for 1.5 h. The extracts were pooled and evaporated in an air-circulating oven at 40°C. Two hundered mL of aliquots from standard sucrose and samples were added to 1 mL of reaction mixture containing 100 mM imidazole buffer (pH 6.9; 40 mM imidazole base, 60 mM imidazole-HCl), 1 mM ATP, 0.5 mM dithiothreitol, 0.4 mM NADP^+^, 0.02% (w/v) BSA, 5 mM MgCl_2_, 20 μg mL^-1^ yeast invertase (EC 3.2.1.26), 2 μg mL^-1^ yeast hexokinase (EC 2.7.1.1) and 1 μg mL^-1^ yeast phospho-glucoisomerase (EC 5.3.1.9). The above mixture was incubated for 30 min at 25°C to allow conversion of glucose and fructose to glucose 6-phosphate. The absorption was recorded at 340 nm before adding 85 μL of glucose-6-phosphate dehydrogenase (70 units mL^-1^) and re-reading after ∼5 min when the absorbance became constant. Simultaneously, blanks were run with 1 mL of the reaction mixture without invertase and with 200 mL of the extract’ (Awasthi et al., 2014). The readings from each sample were converted to sucrose concentrations using a standard curve and expressed as mmol sucrose g^-1^ DW.

Starch concentration was measured using the method of McCreddy et al. (1950). ‘The residue of ethanol extract (prepared as above for sucrose concentration) was washed with 80% ethanol to remove any trace of soluble sugars. Five mL distilled water and 6.5 mL of 52% perchloric acid were added to the residue, which was incubated for 20 min at 0°C. The mixture was then centrifuged and the extract retained. The process mentioned above was repeated 3–4 times and diluted to make 100 mL final volume. To 0.5 mL of diluted extract, 4.5 mL of distilled water was added, followed by addition of 10 mL of chilled anthrone sulphuric acid reagent. The tubes were then heated for 8 min at 100°C in a water bath and cooled to room temperature. The absorbance was recorded at 630 nm. The concentration of starch was calculated from a standard curve plotted with known concentrations of glucose and expressed as μmol g^-1^ DW’ (McCreddy et al., 1950; Awasthi et al., 2014).

To analyze the concentration of reducing sugars, ‘one mL DNSA reagent was added to 1 mL ethanol extract (prepared for sucrose estimation as above). The mixture was heated in a boiling water bath for 12 min and cooled to room temperature before adding 2 mL distilled water. The absorbance was measured at 560 nm against 80% ethanol as a blank rather than ethanol extract. The concentration of reducing sugars was calculated from a standard curve plotted with known concentrations of glucose and expressed as mmol glucose g^-1^ DW’ (Sumner and Howell, 1935; Awasthi et al., 2014).

### Statistical Analysis

The data for the eight genotypes by four treatments and three replicates were analyzed using a one-way analysis of variance (Agristat and Prism statistical software). Treatment correlations were determined using average values per genotype. *Post hoc* test (Tukey’s) to compare means was done using SAS software. Mean values along with standard errors and LSD (*P* < 0.05) values for genotypes, treatments and their interactions are presented in the figures.

## Results

### Phenology

In NS lentil plants, the time to initiate first flowering ranged from 104 to 109 days after sowing (DAS), while the time to initiate first pods ranged from 113–116 DAS (**Tables 2, 7**). In late-sown (LS) plants, flowering, podding and maturity occurred much earlier than the NS plants due to the higher temperatures (**Tables 2, 7**). Drought stress, applied at the 75% podding stage, significantly decreased the days to maturity (9.1–28.1) while heat stress reduced it by 40–56.9 days. The combination of drought and heat stress further reduced the days to maturity (50–73.9; **Tables 2, 7**). Consequently, the duration of flowering and podding was markedly reduced by heat stress (**Table 3**).

**Table 2:** Phenology of eight lentil genotypes contrasting for heat tolerance (HT), heat sensitivity (HS), drought tolerance (DT) and drought sensitivity (DS) in the normal-sown well-watered treatment (Control), normal-sown, drought-stressed treatment (Drought), late-sown well-watered treatment (Heat), and late-sown drought-stressed treatment (Heat+Drought).

Genotype	Control	Drought	Heat	Heat + Drought
**Days to flowering [LSD (genotype × treatment; *P* < 0.001)]: 2.9.**				
DPL 53 (DT1	106.6 ± 2.6a	107.2 ± 2.3a	46.7 ± 2.4b	45.8 ± 2.1b
JL1 (DT2)	107.3 ± 2.4a	108.2 ± 2.1a	44.5 ± 2.1b	46.4 ± 2.4b
ILL 2150(DS1)	105.4 ± 2.1a	107.2 ± 2.4a	48.3 ± 2.2b	46.3 ± 2.3b
ILL 4345(DS2)	108.2 ± 2.6a	106.5 ± 2.2a	46.3 ± 2.3b	47.2 ± 2.5b
1G 2507 (HT1)	106.5 ± 1.9a	108.4 ± 2.5a	47.2 ± 2.2b	48.4 ± 2.1b
1G 4258 (HT2)	107.3 ± 2.1a	109.2 ± 2.3a	43.5 ± 2.5b	45.3 ± 2.4b
1G 3973 (HS1)	109.4 ± 2.3a	106.4 ± 2.1a	45.7 ± 2.3b	47.3 ± 2.2b
1G 3964 (HS2)	108.4 ± 2.4a	106.8 ± 2.2a	46.1 ± 2.5b	47.2 ± 2.6b
**Days to podding [LSD (genotype × treatment; *P* < 0.001)]: 3.1.**				
DPL 53 (DT1	113.4 ± 2.5a	114.2 ± 2.4a	57.8 ± 2.5b	58.2 ± 2.1b
JL1 (DT2)	115.6 ± 2.3a	116.3 ± 2.1a	58.4 ± 2.3b	56.3 ± 2.5b
ILL 2150(DS1)	114.3 ± 2.1a	116.3 ± 2.5a	56.4 ± 2.1b	54.3 ± 2.3b
ILL 4345(DS2)	116.3 ± 2.6a	115.3 ± 2.2a	55.6 ± 2.5b	55.1 ± 2.5b
1G 2507 (HT1)	115.2 ± 2.3a	115.4 ± 2.4a	55.2 ± 2.4b	56.3 ± 2.2b
1G 4258 (HT2)	116.3 ± 2.2a	116.4 ± 2.6a	52.3 ± 2.2b	53.4 ± 2.4b
1G 3973 (HS1)	116.9 ± 2.1a	114.6 ± 2.3a	53.4 ± 2.5b	55.2 ± 2.1b
1G 3964 (HS2)	115.7 ± 2.5a	115.4 ± 2.1a	54.3 ± 2.1b	54.3 ± 2.4b
**Days to maturity [LSD (genotype × treatment; *P* < 0.001)]: 2.8.**				
DPL 53 (DT1	148.4 ± 2.2a	138.8 ± 2.5b	108.4 ± 2.5c	98.3 ± 2.5d
JL1 (DT2)	151.3 ± 2.4a	141.2 ± 2.1b	110.4 ± 2.3c	96.4 ± 2.4d
ILL 2150(DS1)	149.6 ± 2.5a	126.8 ± 2.4c	98.3 ± 2.7d	81.4 ± 2.6f
ILL 4345(DS2)	150.5 ± 2.3a	124.5 ± 2.5c	95.4 ± 2.1d	83.2 ± 2.5f
1G 2507 (HT1)	147.4 ± 2.6a	138.3 ± 2.3b	103.5 ± 2.5c	90.4 ± 2.2e
1G 4258 (HT2)	151.3 ± 2.1a	140.3 ± 2.2b	104.6 ± 2.2c	92.3 ± 2.1e
1G 3973 (HS1)	152.3 ± 2.6a	125.6 ± 2.5c	95.4 ± 2.4d	78.4 ± 2.4g
1G 3964 (HS2)	149.5 ± 2.4a	121.4 ± 2.6c	96.1 ± 2.3d	80.4 ± 2.2fg

*Values are means+SEM. (*n* = 3). Different letters indicate significant differences between means. DT is drought tolerant, DS is drought sensitive, HT is heat tolerant, HS is heat sensitive.*

**Table 3:** Flowering and podding duration in eight lentil genotypes contrasting for heat tolerance (HT), heat sensitivity (HS), drought tolerance (DT) and drought sensitivity (DS) in the normal-sown well-watered treatment (Control), normal-sown drought-stressed treatment (Drought), late-sown well-watered treatment (Heat), and late-sown drought-stressed treatment (Heat + Drought).

Genotype	Control	Drought	Heat	Heat+Drought
**Flowering duration [LSD (genotype × treatment; *P* < 0.001)]: 2.8.**				
DPL 53 (DT1)	34.2 ± 2.1	31.4 ± 2.2 (-2.8)	27.4 ± 1.9 (-6.8)	28.4 ± 1.9 (-5.8)
JL1 (DT2)	31.4 ± 1.8	32.5 ± 2.1 (1.1)	25.8 ± 1.8 (-5.6)	24.3 ± 1.8 (-7.1)
ILL 2150 (DS1)	24.5 ± 1.9	25.6 ± 1.9 (1.1)	19.4 ± 1.7 (-5.1)	18.5 ± 1.6 (-6)
ILL 4345 (DS2)	21.5 ± 1.6	23.4 ± 2.2 (1.9)	18.5 ± 1.8 (-3)	19.3 ± 1.8 (-2.2)
1G 2507 (HT1)	26.3 ± 1.8	27.3 ± 1.8 (1)	19.5 ± 1.9 (-6.8)	18.6 ± 1.9 (-7.7)
1G 4258 (HT2)	24.3 ± 1.8	25.4 ± 1.9 (1.1)	18.3 ± 1.7 (-6)	19.3 ± 1.8 (-5)
1G 3973 (HS1)	20.5 ± 1.9	22.3 ± 2.1 (1.8)	13.5 ± 1.6 (-7)	14.3 ± 1.7 (-6.2)
1G 3964 (HS2)	32.4 ± 2.1	34.5 ± 1.8 (2.1)	24.5 ± 1.8 (-7.9)	22.4 ± 1.8 (-10)
**Podding duration [LSD (genotype × treatment; *P* < 0.001)]: 2.7.**				
DPL 53 (DT1)	27.9 ± 2.1	22.3 ± 2.1 (-5.6)	21.6 ± 1.8 (-6.3)	18.9 ± 1.8 (-9)
JL1 (DT2)	29.3 ± 1.8	24.6 ± 1.9 (-4.7)	22.4 ± 1.9 (-6.9)	19.3 ± 1.9 (-10)
ILL 2150 (DS1)	28.4 ± 1.9	20.4 ± 2.1 (-8)	18.5 ± 1.6 (-9.9)	14.5 ± 1.7 (-13.9)
ILL 4345 (DS2)	27.1 ± 1.9	20.6 ± 1.9 (-6.5)	17.8 ± 1.6 (-9.3)	14.2 ± 1.6 (-12.9)
1G 2507 (HT1)	28.3 ± 2.1	23.4 ± 2.1 (-4.9)	21.4 ± 2.1 (-6.9)	19.5 ± 1.7 (-8.8)
1G 4258 (HT2)	29.5 ± 2.2	24.3 ± 1.8 (-5.2)	20.6 ± 1.9 (-8.9)	18.4 ± 1.6 (-11.1)
1G 3973 (HS1)	27.9 ± 2.4	20.1 ± 1.9 (-7.8)	17.8 ± 1.7 (-10.1)	14.6 ± 1.5 (-13.3)
1G 3964 (HS2)	28.1 ± 2.1	20.8 ± 1.7 (-7.3)	18.4 ± 1.8 (-9.7)	15.3 ± 1.7 (-12.8)

*Values are means+SEM. (*n* = 3). Different letters indicate significant differences between means. Values in Parenthesis indicate difference in days, compared to control. DT is drought tolerant, DS is drought sensitive, HT is heat tolerant, HS is heat sensitive.*

### Growth and Yield

**FIGURE 2:**
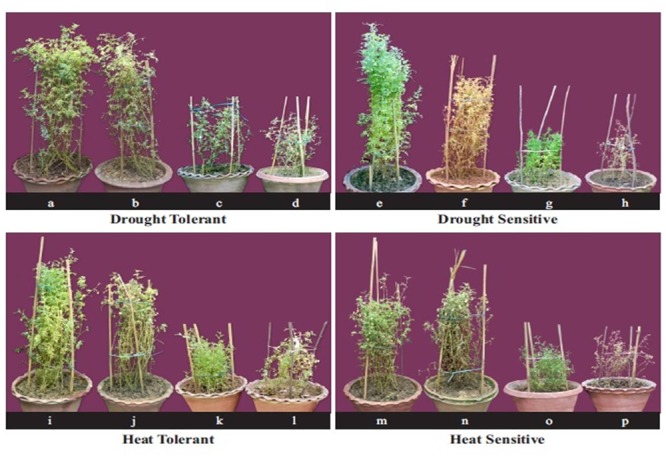
The effect of drought and heat stress, and their combination on biomass in tolerant and sensitive lentil genotypes. For the drought tolerant genotype (DPL 53) (a) well-watered, (b) drought-stressed, (c) heat-stressed, (d) heat + drought. For the drought-sensitive genotype (ILL 2150) (e) well-watered, (f) drought-stressed, (g) heat-stressed, (h) heat + drought. For the heat tolerant genotype (1G 2507) (i) well-watered, (j) drought-stressed, (k) heat-stressed, (1) heat + drought. For the heat sensitive genotype (1G 3973). (m) well-watered, (n) drought-stressed, (o) heat-stressed, (p) heat + drought.

In the NS control plants, the aboveground biomass (shoots and pod shells) ranged from 5.54 to 6.48 g plant^-1^ across the four genotypes (**Tables 4, 7** and **Figure 2**). Drought stress reduced the aboveground biomass by 27–29% in HT genotypes, 33–36% in HS genotypes, 29–31% in DT genotypes and 36–41% in DS genotypes. Heat stress in the LS plants reduced the aboveground biomass more than drought stress (42–53% vs. 27–41%). Relative to the NS control plants, heat stress reduced biomass by 42–46% in HT genotypes, 48–54% in HS genotypes, 43–46% in DT genotypes, and 47–50% in DS genotypes. The combined drought and heat stress treatment reduced the aboveground biomass by 59–67% in tolerant genotypes and 70–78% in sensitive genotypes.

**Table 4:** Aboveground biomass, pod number per plant and pod weight per plant in eight lentil genotypes contrasting for heat tolerance (HT), heat sensitivity (HS), drought tolerance (DT) and drought sensitivity (DS) in the normal-sown well-watered treatment (Control), normal-sown drought-stressed treatment (Drought; D), late-sown well-watered treatment (Heat; H), and late-sown drought-stressed treatment (Heat + Drought; H + D).

	Biomass				Pod number per plant				Pod weight per plant			
Genotype	Control	D	H	H + D	Control	D	H	H + D	Control	D	H	H + D
DPL 53 (DT1)	5.79 ± 0.63a	4.11 ± 0.62b (29%)	3.12 ± 0.65c (46.1%)	1.91 ± 0.45d (67%)	149.3 ± 6.7a	64.1 ± 5.7c (57%)	40.4 ± 2.9cd (72.9%)	26.8 ± 2.1de (82%)	4.11 ± 0.31a	1.72 ± 0.18c (58.1%)	1.47 ± 0.15d (64.2%)	0.82 ± 0.12e (80%)
JL1 (DT2)	5.89 ± 0.74a	3.98 ± 0.67b (32.4%)	2.91 ± 0.63c (50.5%)	2.97 ± 0.41c (49.5%)	142.3 ± 7.2b	71.5 ± 5.8c (49.7%)	42.9 ± 2.7cd (69.8%)	28.4 ± 2.3de (80%)	3.79 ± 0.26a	1.78 ± 0.19c (53%)	1.41 ± 0.17d (62.7%)	0.68 ± 0.11e (82%)
ILL 2150 (DS1)	6.11 ± 0.71a	3.91 ± 0.71b (36%)	3.36 ± 0.66bc (45%)	1.40 ± 0.32e (77%)	155.3 ± 6.9a	46.5 ± 4.9cd (70%)	26.4 ± 1.7de (83%)	13.9 ± 1.1e (91%)	4.30 ± 0.31a	1.20 ± 0.17d (72%)	0.77 ± 0.15e (82%)	0.34 ± 0.09f (92%)
ILL 4345 (DS2)	5.83 ± 0.69a	3.42 ± 0.63b (41.3%)	3.70 ± 0.69b (36.5%)	1.52 ± 0.25e (73.9%)	138.9 ± 7.4b	40.2 ± 4.1cd (71%)	30.5 ± 1.8de (78%)	17.6 ± 1.3e (87.3%)	3.6 ± 0.29a	0.93 ± 0.14de (74.1%)	0.72 ± 0.16e (80%)	0.31 ± 0.06f (91.3%)
1G 2507 (HT1)	6.31 ± 0.82a	4.60 ± 0.71ab (27%)	4.98 ± 0.63ab (21%)	2.41 ± 0.43d (61.8%)	148.2 ± 6.9a	62.2 ± 4.6cd (58%)	44.2 ± 2.1cd (70.1%)	31.2 ± 2.3de (78.9%)	4.11 ± 0.25a	2.54 ± 0.19b (38.1%)	1.32 ± 0.15d (67.8%)	0.69 ± 0.08e (83.2%)
1G 4258 (HT2)	6.48 ± 0.83a	4.56 ± 0.66ab (29.6%)	3.45 ± 0.67bc (46.7%)	2.26 ± 0.32d (65.1%)	151.3 ± 7.7a	57.4 ± 4.8cd (62%)	42.3 ± 1.8cd (72%)	25.7 ± 1.4de (83%)	4.06 ± 0.25a	2.46 ± 0.16b (39.4%)	1.28 ± 0.13d (68.4%)	0.56 ± 0.08e (86.2%)
1G 3973 (HS1)	5.76 ± 0.76a	3.66 ± 0.68bc (36.4%)	2.67 ± 0.63cd (53.6%)	1.38 ± 0.25e (76%)	142.3 ± 6.8b	43.1 ± 4.2d (69.7%)	27.5 ± 1.4de (80.6%)	11.9 ± 1.8e (91.6%)	3.91 ± 0.26a	1.17 ± 0.17de (70%)	0.66 ± 0.12e (83.1%)	0.35 ± 0.09f (91%)
1G 3964 (HS2)	5.54 ± 0.72ab	3.75 ± 0.64bc (32.3%)	2.93 ± 0.59cd (47.1%)	1.66 ± 0.28e (70%)	136.4 ± 7.3b	41.2 ± 4.4d (69.7%)	22.8 ± 1.4de (83.2%)	10.3 ± 1.3e (92.4%)	3.56 ± 0.28ab	0.99 ± 0.12de (72.1%)	0.71 ± 0.11e (80%)	0.28 ± 0.06f (92.1%)

*LSD values (genotype × treatment; *P* < 0.0001) for biomass = 0.91, pod no./plant = 7.9 and pod weight/plant = 0.19 Values are the means + SEM. (*n* = 3). Different letters indicate significant differences between means. Values in Parenthesis indicate percentage reduction over control. DT is drought tolerant, DS is drought sensitive, HT is heat tolerant, HS is heat sensitive.*

Heat stress reduced pod numbers (70–84%) more than drought stress (50–71%) but less than the combined stress (80–91%; **Tables 4, 7**). Heat stress reduced pod numbers by 70–72% in HT genotypes, 69–75% in DT genotypes, and 80–84% in the DS and HS genotypes. Drought stress reduced pod numbers by 58–60% in HT genotypes, 50–55% in DT genotype and 68–71% in sensitive genotypes. The combined stress treatment reduced pod numbers less in the tolerant genotypes (78–82%) than the sensitive genotypes (90–92%).

Pod weights decreased more with heat stress (73–81%) than drought stress (55–77%), while the combined stress resulted in a 78–90% reduction (**Tables 4, 7**). Heat stress reduced pod weights by 73–74% in HT genotypes, 70–75% in DT genotypes, and 81–83% in DS and HS genotypes; the corresponding values for drought stress were 60–62%, 50–53%, and 68–71%, compared with the NS control plants. The combined stress treatment reduced pod weights by 80–86% in tolerant genotypes and 92–93% in sensitive genotypes.

Seed numbers in NS control plants ranged from 141–168 plant^-1^, which declined to 20–54 in heat-stressed plants, 36–68 in drought-stressed plants and 9–44 in the combined heat and drought stress treatment (**Tables 5, 7**). Drought stress reduced seed numbers by 60–65% in HT genotypes, 70–73% in HS genotypes, 55–56% in DT genotypes, 76–77% in DS genotypes. Heat stress reduced seed numbers by 63–65% in HT genotypes, 86–88% in HS genotypes, 62–67% in DT genotypes, and 81–86% in DS genotypes. The combined stress reduced seed numbers by 78–80% in HT genotypes, 71–73% in DT genotypes, and 90–92% in the HS and DS genotypes.

**Table 5:** Seed number per plant, seed weight per plant and individual seed weight in eight lentil genotypes contrasting for heat tolerance (HT), heat sensitivity (HS), drought tolerance (DT) and drought sensitivity (DS) in the normal-sown well-watered treatment (Control), normal-sown drought-stressed treatment (Drought; D), late-sown well-watered treatment (Heat; H), and late-sown drought-stressed treatment (Heat + Drought; H + D).

	Seed number per plant				Seed weight (g per plant)				Individual seed weight (g)			
Genotype	Control	D	H	H + D	Control	D	H	H + D	Control	D	H	H + D
DPL 53 (DT1)	154.2 ± 5.8a	68.1 ± 4.2c (55.8%)	50.3 ± 3.3d (67.3%)	44.6 ± 2.6d (71%)	3.34 ± 0.16a	1.52 ± 0.17c (54.4%)	1.14 ± 0.13d (65.8%)	0.93 ± 0.11d (72.1%)	0.025 ± 0.0013a	0.019 ± 0.0011b (24%)	0.022 ± 0.0011ab (12%)	0.017 ± 0.0013b (32%)
JL1 (DT2)	145.2 ± 5.1b	62.5 ± 4.4c (56.9%)	54.3 ± 2.8d (62.6%)	39.1 ± 2.5d (73%)	3.09 ± 0.18b	1.56 ± 0.16c (49.5%)	1.12 ± 0.11d (63.7%)	0.83 ± 0.09de (73.1%)	0.023 ± 0.0014ab	0.019 ± 0.0012b (17.3%)	0.020 ± 0.0012ab (13%)	0.016 ± 0.0011b (30.4%)
ILL 2150 (DS1)	168.3 ± 5.9a	40.3 ± 4.1e (76%)	26.4 ± 2.1f (84.3%)	13.8 ± 1.3g (91.8%)	3.79 ± 0.17a	1.02 ± 0.17d (73%)	0.63 ± 0.09e (83.3%)	0.33 ± 0.07e (91.2%)	0.023 ± 0.0012ab	0.015 ± 0.0011cd (34.7%)	0.017 ± 0.0012cd (26%)	0.010 ± 0.0012e (56.5%)
ILL 4345 (DS2)	159.7 ± 5.3b	36.2 ± 3.2e (77.3%)	29.3 ± 2.3f (81.6%)	12.7 ± 1.2g (92%)	3.18 ± 0.16b	0.76 ± 0.14de (76.1%)	0.58 ± 0.09e (81.7%)	0.30 ± 0.08e (90.5%)	0.015 ± 0.0011cd	0.013 ± 0.0011de (13.3%)	0.013 ± 0.0012de (13.3%)	0.0083 ± 0.0011e (46.6%)
1G 2507 (HT1)	152.4 ± 5.7a	62.3 ± 3.6c (59.1%)	53.1 ± 2.2d (65.1%)	33.4 ± 2.2d (78%)	3.54 ± 0.18a	1.55 ± 0.15c (56.2%)	1.06 ± 0.09d (70%)	1.06 ± 0.06d (70%)	0.020 ± 0.0012b	0.016 ± 0.0011bc (20%)	0.018 ± 0.0012b (10%)	0.012 ± 0.0012bc (40%)
1G 4258 (HT2)	160.3 ± 5.5a	56.1 ± 3.1c (65%)	56.7 ± 2.4c (64.6%)	31.76 ± 2.1d (80.2%)	3.43 ± 0.14a	1.34 ± 0.15c (60.9%)	1.10 ± 0.10d (67.9%)	1.03 ± 0.07d (69.9%)	0.020 ± 0.0011b	0.016 ± 0.0011bc (20%)	0.017 ± 0.0012b (15%)	0.011 ± 0.0011bc (45%)
1G 3973 (HS1)	148.6 ± 5.8b	40.5 ± 3.5de (72.7%)	20.8 ± 2.1ef (86%)	13.3 ± 1.4g (91%)	3.28 ± 0.13b	0.74 ± 0.16de (77.4%)	0.37 ± 0.06e (88.7%)	0.20 ± 0.04e (93.9%)	0.016 ± 0.0012cd	0.012 ± 0.0012de (25%)	0.013 ± 0.0011de (18.75%)	0.009 ± 0.0011e (43.75%)
1G 3964 (HS2)	141.5 ± 5.3b	42.3 ± 3.6de (70.1%)	20.3 ± 2.3ef (85.6%)	8.96 ± 0.89g (93.6%)	3.18 ± 0.14b	0.69 ± 0.13de (78.3%)	0.30 ± 0.07e (90.5%)	0.16 ± 0.03e (94.9%)	0.019 ± 0.0012b	0.013 ± 0.0011de (31.5%)	0.016 ± 0.0012bc (15.7%)	0.010 ± 0.0012e (47.3%)

*LSD values (genotype × treatment; *P* < 0.001) for seed no./plant = 6.1, seed weight/plant = 0.19 and individual seed weight = 0.0018. Values are means + SEM. (*n* = 3). Different letters indicate significant differences between means. Values in Parenthesis indicate percentage reduction over control. DT is drought tolerant, DS is drought sensitive, HT is heat tolerant, HS is heat sensitive.*

**Table 6:** Correlation coefficients of various traits with biomass and seed weight/plant under combined heat and drought stress.

	Heat + Drought	
Trait	Biomass	Seed weight per plant
Pod weight per plant	0.700	0.957
Pod number per plant	0.837	0.971
Seed number per plant	0.748	0.966
Individual seed weight	0.647	0.783
Membrane damage	–0.787	–0.987
Stomatal conductance	0.780	0.907
PSII function	0.775	0.901
Photosynthetic rate	0.761	0.953
Chlorophyll	0.673	0.851
RuBisCo activity	0.607	0.902
Osmotic potential	0.814	0.986
RLWC	0.861	0.967
Sucrose concentration (leaf)	0.814	0.804
Sucrose concentration (seed)	0.860	0.908
Sucrose synthase activity (leaf)	0.308	0.601
Sucrose synthase activity (seed)	0.832	0.927
Acid invertase activity (leaf)	0.107	0.559
Acid invertase activity (seed)	0.557	0.817
Reducing sugars concentration (leaf)	0.820	0.875
Reducing sugars concentration (seed)	0.780	0.956
Starch concentration (leaf)	0.583	0.568
Starch concentration (seed)	0.771	0.833
Starch phosphorylase activity (leaf)	0.753	0.867
Starch phosphorylase activity (seed)	0.494	0.478
β-amylase activity (leaf)	–0.243	–0.246
β-amylase activity (seed)	0.716	0.821

**Table 7:** Analysis of variance (ANOVA) showing the level of statistical significance of the traits measured in eight lentil genotypes (Genotype) given four treatments [(i) normal-sown well-watered, (ii) normal-sown drought-stressed, (iii) late-sown well-watered, and (iv) late-sown drought-stressed (Treatment)] and the significance of the interaction of Genotype × Treatment (Interaction)].

	Genotype	Treatment	Interaction
Days to flowering	^∗∗∗^	^∗∗∗^	^∗^
Days to podding	^∗∗∗^	^∗∗∗^	^∗^
Days to maturity	^∗∗∗^	^∗∗∗^	^∗∗^
Flowering duration	^∗∗∗^	^∗∗∗^	^∗^
Podding duration	^∗∗∗^	^∗∗∗^	^∗∗^
Aboveground biomass/plant	^∗∗∗^	^∗∗∗^	^∗∗∗^
Pod number/plant	^∗∗∗^	^∗∗∗^	^∗∗∗^
Pod weight/plant	^∗∗∗^	^∗∗∗^	^∗∗∗^
Seed number/plant	^∗∗∗^	^∗∗∗^	^∗∗∗^
Individual seed weight	^∗∗^	^∗∗^	^∗∗∗^
Membrane damage	^∗∗^	^∗∗∗^	^∗∗∗^
Stomatal conductance	^∗∗^	^∗∗∗^	^∗∗∗^
PSII function	^∗∗∗^	^∗∗∗^	^∗∗∗^
Photosynthetic rate	^∗∗∗^	^∗∗∗^	^∗∗∗^
Chlorophyll concentration	^∗∗∗^	^∗∗∗^	^∗∗∗^
RuBisCo activity	^∗∗∗^	^∗∗∗^	^∗∗∗^
Osmotic potential	^∗∗∗^	^∗∗∗^	^∗∗∗^
Photosynthetic rate	^∗∗∗^	^∗∗∗^	^∗∗∗^
Relative leaf water content	^∗∗∗^	^∗∗∗^	^∗∗∗^
Sucrose concentration (leaf)	^∗∗^	^∗∗∗^	^∗∗∗^
Sucrose concentration (seed)	^∗∗^	^∗∗∗^	^∗∗∗^
Sucrose synthase activity (leaf)	^∗∗^	^∗∗∗^	^∗∗∗^
Sucrose synthase activity (seed)	^∗∗^	^∗∗∗^	^∗∗∗^
Acid invertase activity (leaf)	^∗∗^	^∗∗∗^	^∗∗∗^
Acid invertase activity (seed)	^∗^	^∗∗∗^	^∗∗∗^
Reducing sugars concentration (leaf)	^∗^	^∗∗∗^	^∗∗∗^
Reducing sugars concentration (seed)	^∗∗^	^∗∗∗^	^∗∗∗^
Starch concentration (leaf)	^∗∗^	^∗∗∗^	^∗∗∗^
Starch concentration (seed)	^∗∗^	^∗∗∗^	^∗∗∗^
Starch phosphorylase activity (leaf)	^∗∗^	^∗∗∗^	^∗∗∗^
Starch phosphorylase activity (seed)	^∗∗^	^∗∗∗^	^∗∗∗^
β-amylase activity (leaf)	^∗∗^	^∗∗∗^	^∗∗∗^
β-amylase activity (seed)	^∗∗^	^∗∗∗^	^∗∗∗^

*^∗^*P* ≤ 0.05; ^∗∗^*P* ≤ 0.01; ^∗∗∗^*P* ≤ 0.001, n.s., not significant.*

**FIGURE 3:**
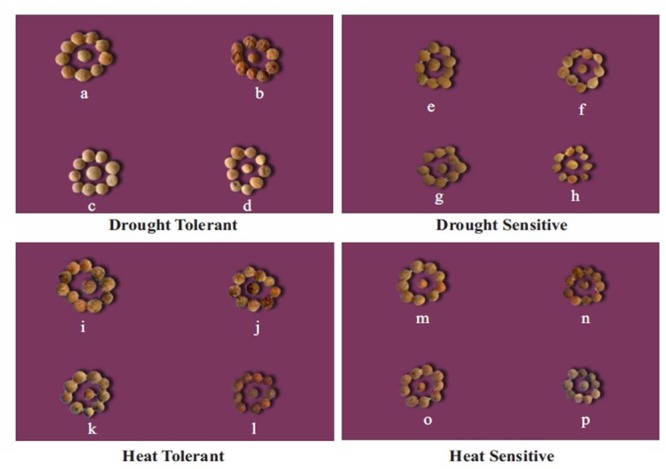
Effect of drought and heat stress, and their combination on seed size in tolerant and sensitive lentil genotypes. For the drought tolerant genotype (DPL 53): (a) well-watered, (b) drought-stressed, (c) heat-stressed, (d) heat + drought. For the drought-sensitive genotype (ILL 2150) (e) well-watered, (f) drought-stressed, (g) heat-stressed, (h) heat + drought. For the heat tolerant genotype (1G 2507) (i) well-watered, (j) drought-stressed, (k) heat-stressed, (l) heat + drought. For the heat sensitive genotype (1G 3973) (m) well-watered, (n) drought-stressed, (o) heat-stressed, (p) heat + drought stressed in heat sensitive genotype (1G 3973).

In the NS control plants, seed weights ranged from 3.18–3.79 g plant^-1^ (**Tables 5, 7** and **Figure 3**). Heat stress reduced seed weights (71–90%) more than drought stress (50–78%). Drought stress reduced seed weights by 56–59% in HT genotypes, 77–78% in HS genotypes, 49–54% in DT genotypes, and 73–76% in DS genotypes. Heat stress reduced seed weights by 65–68% in HT genotypes 89–90% in HS genotypes, 63–65% in DT genotypes, and 81–83% in DS genotypes. The combined stress reduced seed weights by 70–76% in HT genotypes, 93–95% in HS genotypes, 71–73% in DT genotypes, and 90–91% in DS genotypes (**Tables 5, 7** and **Figure 3**).

In the NS control plants, individual seed weights ranged from 16 to 25 mg, which decreased by 20–34% in drought-stressed plants and 12–26% in heat-stressed plants (**Table 5, Figure 3**). The combined drought and heat stress treatment reduced individual seed weights by 40–45% in HT genotypes, 30–32% in DT genotypes, and 44–56% in the HS and DS genotypes (**Tables 5, 7** and **Figure 3**).

### Leaf Water Status and Membrane Damage

**FIGURE 4:**
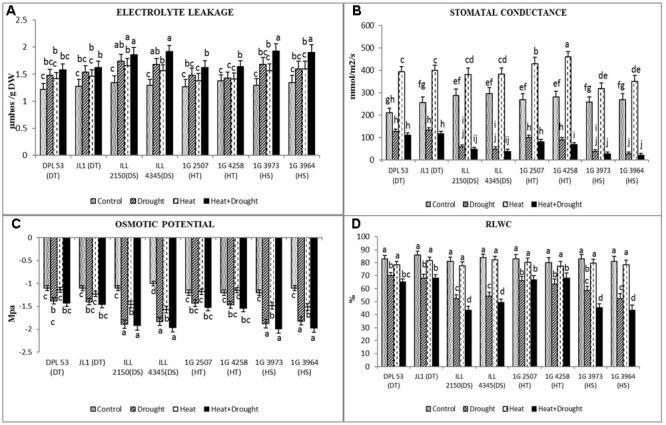
**(A)** Electrolyte leakage, **(B)** stomatal conductance, **(C)** osmotic potential and **(D)** relative leaf water content (RLWC) in eight lentil genotypes contrasting for heat tolerance (HT), heat sensitivity (HS), drought tolerance (DT) and drought sensitivity (DS) in the NS well-watered treatment (Control), normal-sown drought-stressed treatment (Drought), late-sown well-watered treatment (Heat), and late-sown drought-stressed treatment (Heat + Drought). LSD values (genotype × treatment) for **(A)** electrolyte leakage = 0.31, **(B)** stomatal conductance = 21.2, **(C)** osmotic potential = 0.21 and **(D)** RLWC = 3.9. Values are means + SEM. (*n* = 3). Different letters on the vertical bars indicate significant differences between means.

Membrane damage to leaf tissue was assessed by measuring electrical conductivity (**Figure 4** and **Table 7**). Drought stress damaged membranes more (21–40%) than heat stress (14.2–30%), compared with the NS control plants, with more impact on sensitive genotypes. The combined drought and heat stress increased this damage to 33–60%. The DT genotypes had significantly less (20–26%) tissue damage than the sensitive genotypes (38–60%).

Drought stress reduced leaf water status, measured as RLWC, more than heat stress (1.25–1.54-fold vs. 1.02–1.05-fold) but less so than the combined stress (1.38–1.86 fold; **Figure 4** and **Table 7**). The tolerant genotypes maintained higher RLWC (65–68%) than the sensitive genotypes (43–49%) in the presence of combined stresses.

In the NS control plants, leaf osmotic potential ranged from -1.1 to -1.2 MPa (**Figure 4** and **Table 7**). Drought stress reduced this -1.38 to -1.4 MPa in tolerant genotypes and -1.82 to -1.89 MPa in sensitive genotypes. The reduction was relatively less under heat stress. Under the combined stress, the osmotic potential was relatively higher in tolerant genotypes (-1.43 to -1.54 MPa) than the sensitive genotypes (-1.92 to -1.99 MPa).

In the NS control plants, stomatal conductance (*g*s) ranged from 210 to 295 mmol m^-2^ s^-1^ (**Figure 4** and **Table 7**). Drought stress reduced *g*s by 40–90%, averaged across all genotypes, while heat stress increased *g*s by 21–64%. The combined heat and drought stress treatment reduced *g*s by 47–92%, with the DT genotypes showing less reduction (47–54%), compared to the other genotypes (70–92% reduction).

### Photosynthetic Function

**FIGURE 5:**
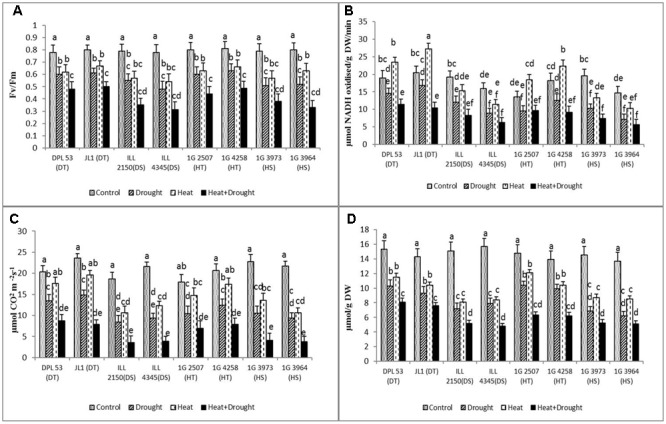
**(A)** PSII function, **(B)** RuBisCo, **(C)** photosynthetic activity and **(D)** chlorophyll concentration of eight lentil genotypes contrasting for heat tolerance (HT), heat sensitivity (HS), drought tolerance (DT) and drought sensitivity (DS) in the normal-sown well-watered treatment (Control), normal-sown drought-stressed treatment (Drought), late-sown well-watered treatment (Heat), and late-sown drought-stressed treatment (Heat + Drought). LSD values (genotype × treatment) for **(A)** PSII function = 0.06, **(B)** RuBiSCo = 2.4, **(C)** photosynthetic activity = 2.1 and **(D)** chlorophyll concentration = 0.39. Values are means + SEM. (*n* = 3). Different letters on the bars indicate significant differences between means.

The damage to photosynthetic efficiency (**Figure 5** and **Table 7**) was measured as a change in Fv/Fm ratio, which did not vary significantly among NS plants (0.78–0.80), but decreased more under drought stress (0.51–0.63) than heat stress (0.57–0.67). Under the combined stress, tolerant genotypes had significantly higher chlorophyll fluorescence (0.44–0.48) than sensitive genotypes (0.31–0.33).

Heat stress significantly increased RuBisCo activity in tolerant genotypes (22–32%), compared to the NS control plants (**Figure 5** and **Table 7**). In contrast, drought stress significantly decreased RuBisCo activity, more so in the sensitive genotypes (37–51.9% reduction). The combined stress severely inhibited RuBisCo activity in all genotype (45–85% reduction); DT genotypes maintained the highest activity (10.4–11.5 μmol NADH oxidized g^-1^ DW min^-1^), followed by HT genotypes (9.1–9.6 μmol NADH oxidized g^-1^ DW min^-1^).

The photosynthetic rate (Pn) decreased significantly more under drought (33.4–56.6%) than heat stress (13.3–43%), compared to the NS control plants (**Figure 5** and **Table 7**). Under the combined stress, Pn declined to its lowest values (57–82% reduction), less so in the HT genotypes (6.9–7.9 μmol CO_2_ m^-2^ s^-1^) and DT genotypes (7.9–8.7 μmol CO_2_ m^-2^ s^-1^) compared to sensitive genotypes (3.5–4.1 μmol CO_2_ m^-2^ s^-1^).

The leaf chlorophyll (Chl) concentration of the NS control plants varied slightly between genotypes (13.7–15.7 μmol g^-1^ DW) (**Figure 5** and **Table 7**). Drought stress reduced the Chl concentration (29.7–54.4%) more than heat stress (25.1–39.4%). The combined stress further reduced Chl concentration in all genotypes (46.8–78.9%), less so in the HT genotypes (6.3–7.5 μmol g^-1^ DW) and DT genotypes (7.6–8.1 μmol g^-1^ DW) than the sensitive genotypes (4.9–5.2 μmol g^-1^ DW).

### Sucrose Metabolism in Leaves and Seeds

**FIGURE 6:**
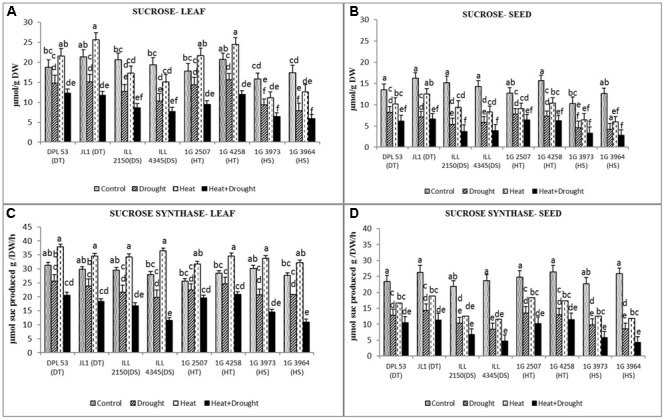
Sucrose content in **(A)** leaf and **(B)** seed and sucrose synthase activity in **(C)** leaf and **(D)** seed in eight lentil genotypes contrasting for heat tolerance (HT), heat sensitivity (HS), drought tolerance (DT) and drought sensitivity (DS) in the normal-sown well-watered treatment (Control), normal-sown drought-stressed treatment (Drought), late-sown well-watered treatment (Heat), and late-sown drought-stressed treatment (Heat + Drought). LSD values (genotype × treatment) for **(A)** sucrose in leaf = 2.1, **(B)** sucrose in seed = 1.9, **(C)** sucrose synthase activity in leaf = 2.4, **(D)** sucrose synthase activity in seed = 1.8. Values are means + SEM (*n* = 3). Different letters on the vertical bars indicate significant differences between means.

Sucrose synthesized in the leaves and seeds are used for seed filling. Hence, both these organs were tested for endogenous sucrose concentration and the enzymes associated with its synthesis. In the NS control plants, the sucrose concentration ranged from 15.8 to 20.7 μmol g^-1^ DW in leaves and 12.7–16.2 μmol g^-1^ DW in seeds (**Figure 6** and **Table 7**). Drought stress reduced this to 7.9–15.7 μmol g^-1^ DW (19.6–54.5%) in leaves but heat stress increased it to 11.1–25.6 μmol g^-1^ DW (14.9–21.3%). The combined stress reduced the sucrose concentration to 5.9–11.7 μmol g^-1^ DW (34.2–66%) across the genotypes. In seeds, drought stress reduced sucrose concentration more (4.2–8.2 μmol g^-1^ DW) (38.5–66.6%) than heat stress (6.4–12.5 μmol g^-1^ DW) (22.8–53.1%) (**Figure 6** and **Table 7**). The combined stress reduced sucrose concentrations further (2.8–6.6 μmol g^-1^ DW) (54.8–76%), less so in HT genotypes (5.1–6.2 μmol g^-1^ DW) (60.5–73.3%) and DT gentoypes (5.5–6.2 μmol g^-1^ DW) (54.8–59.2%) than the sensitive genotypes (2.8–3.9 μmol g^-1^ DW) (67.9–76%).

Sucrose phosphate synthase activity is a marker for sucrose-synthesizing capacity. In leaves, drought stress decreased the activity of this enzyme by 13–31% while heat stress increased it by 16–30% (**Figure 6** and **Table 7**). The combined stress reduced enzyme activity by 33–60%. In seeds, drought stress decreased sucrose phosphate synthase activity more (45–67%) compared to heat stress (28–51%) and the combined stress inhibited it further (57–83%). The tolerant genotypes were able to retain higher enzyme activity (8.7–11.3 units) than the sensitive genotypes (4.3–6.8 units).

**FIGURE 7:**
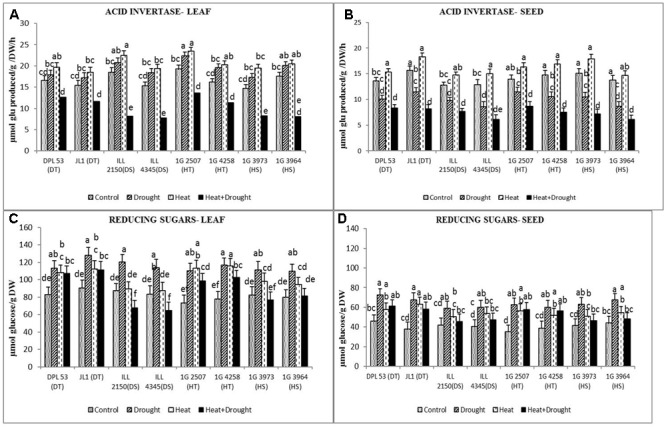
Add invertase activity in **(A)** leaf and **(B)** seed and reducing sugars concentration in **(C)** leaf and **(D)** seed in eight lentil genotypes contrasting for heat tolerance (HT), heat sensitivity (HS), drought tolerance (DT) and drought sensitivity (DS) in the normal-sown well-watered treatment (Control), normal-sown drought-stressed treatment (Drought), late-sown well-watered treatment (Heat), and late-sown drought-stressed treatment (Heat + Drought). LSD values (genotype × treatment) for **(A)** acid invertase activity in leaf = 2.2, **(B)** acid invertase activity in seed = 1.7, **(C)** reducing sugars concentration in leaf = 8.3, **(D)** reducing sugar concentration in seed = 7.4. Values are means + SEM. (*n* = 3). Different letters on the vertical bars indicate significant differences between means.

Acid invertase converts sucrose to glucose and fructose, the reducing sugars. In leaves, the activity of this enzyme increased more under heat stress (18–34%) than drought stress (7.8–24.6%) (**Figure 7** and **Table 7**). The combined stress reduced acid invertase activity by 22–49%, more so in HS genotypes (35–49%) than the tolerant genotypes (29–30%). In seeds, drought stress reduced acid invertase activity by 17–36% while heat stress increased it by 6–17%. The combined stress further reduced acid invertase activity (38–55.7%), more so in DT genotypes than the sensitive genotypes.

In leaves, drought stress increased the concentration of reducing sugars more (36–56%) than heat stress (11.3–24.4%), especially in HT and DT genotypes (**Figure 7** and **Table 7**). The combined stress increased the concentration of reducing sugars to higher than the NS control plants, especially in the HT genotypes (99–102 μmol glucose g^-1^ DW) and DT genotypes (107–111 μmol glucose g^-1^ DW) compared to the sensitive genotypes (64–81 μmol glucose g^-1^ DW). In seeds, drought stress increased the concentration of reducing sugars more (41–78%) than heat stress (26–66%). The combined stress increased this further, more so in tolerant genotypes (56–61 μmol glucose g^-1^ DW) than sensitive genotypes (46–47 μmol glucose g^-1^ DW).

### Starch Metabolism in Leaves and Seeds

**FIGURE 8:**
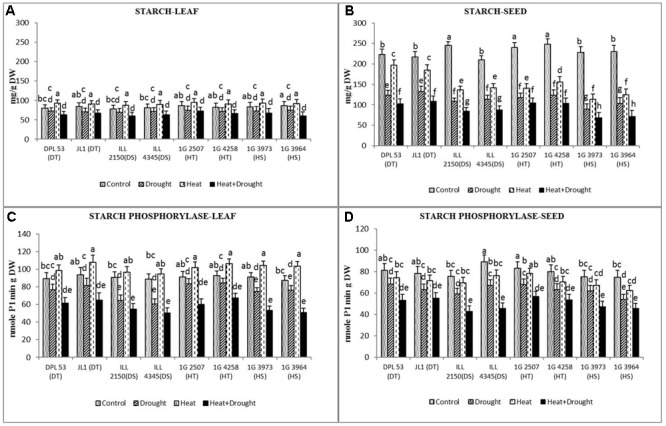
Starch concentration in **(A)** leaf and **(B)** seed and starch phosphorylation activity in **(C)** leaf and **(D)** seed in eight lentil genotypes contrasting for heat tolerance (HT), heat sensitivity (HS), drought tolerance (DT) and drought sensitivity (DS) in the normal-sown well-watered treatment (Control), normal-sown drought-stressed treatment (Drought), late-sown well-watered treatment (Heat), and late-sown drought-stressed treatment (Heat + Drought). LSD values (genotype × treatment) for **(A)** starch concentration in leaf = 15.4, **(B)** starch concentration in seed = 12.4, **(C)** starch phosphorylation activity in leaf = 7.7, **(D)** starch phosphorylation activity in seed = 6.9. Values are means + SEM. (*n* = 3). Different letters on the vertical bars indicate significant differences between means.

Heat stress increased the starch concentration in leaves (6.9–16%) but not in seeds (11.3–50% reduction) while drought stress reduced the starch concentration in both leaves (10–16%) and seeds (37–60%) (**Figure 8** and **Table 7**). The combined stress further decreased the starch concentration in leaves (16–30%) and seeds (50–70%). Tolerant genotypes retained significantly more starch (96–102.4 μmol g^-1^ DW) in their seeds than sensitive genotypes (68.4–93.4 μmol g^-1^ DW).

Heat stress increased the activity of starch phosphorylase (involved in starch synthesis) in the leaves (9.9–18.4%) but drought stress decreased it by 8.8–31% (**Figure 8** and **Table 7**). In seeds, the activity of starch phosphorylase declined by 7.9–16.3% under heat stress and by 16–24% under drought stress. The combined stress reduced the enzyme activity by 30–42% in leaves and by 31–48% in seeds, less so in the HT and DT genotypes.

**FIGURE 9:**
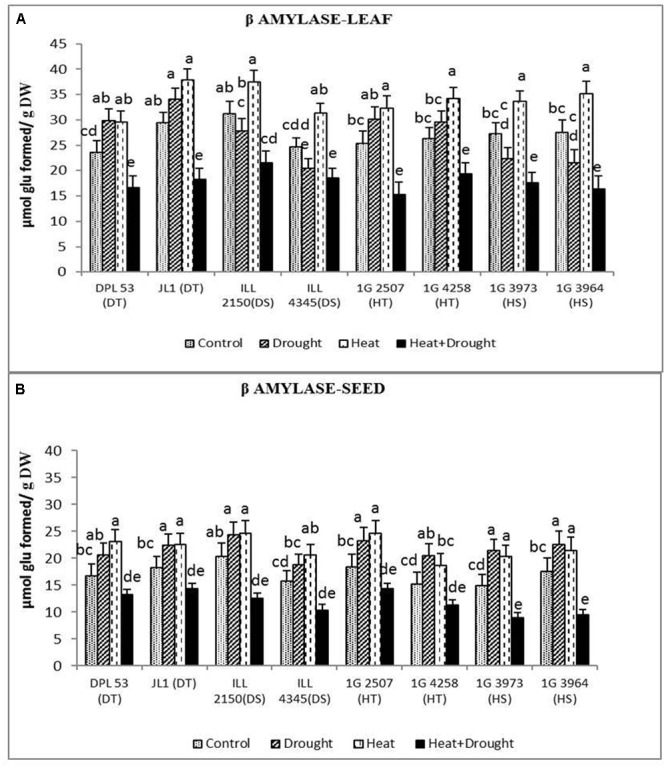
β amylase activity in **(A)** leaf and **(B)** seed in eight lentil genotypes contrasting for heat tolerance (HT), heat sensitivity (HS), drought tolerance (DT) and drought sensitivity (DS) in the normal-sown well-watered treatment (Control), normal-sown drought-stressed treatment (Drought), late-sown well-watered treatment (Heat), and late-sown drought-stressed treatment (Heat + Drought). LSD values (genotype × treatment) for **(A)** β amylase activity in leaf = 2.7, and **(B)** β amylase activity in seed = 2.2. Values are means + SEM. (*n* = 3). Different letters on the vertical bars indicate significant differences between means.

In leaves, the activity of β-amylase (converts starch into sugars) increased under heat stress (19.8–30.0%) and drought stress (10.9–21.4%), but the combined stress decreased it by 24–40% (**Figure 9** and **Table 7**). In seeds, β-amylase activity increased by 19–38% under heat stress and 18–43% under drought stress, but decreased it by 20–45% under the combined stress, more so in the sensitive genotypes.

## Discussion

Delayed sowing is a commonly used practice in parts of the world where crops are grown in the cooler months to expose the developing pods and seeds to high temperatures, but the confounding effects of some other environmental variables such as relative humidity and photoperiod cannot be completely ruled out. Nevertheless, this technique has been used to assess the heat stress responses of chickpea (Krishnamurthy et al., 2011; Upadhyaya et al., 2011), lentil (Bhandari et al., 2016; Sita et al., 2017) and mungbean (Sharma et al., 2016). However, high temperatures and related low VPDs enhance the transpiration rate, and consequently the decreasing soil moisture can impose both water as well as heat stress. Hence, to assess the effects of heat stress, frequent irrigation of the plants is required, which was done in the present study, except in plants which were drought-stressed at the time of seed filling. Photoperiod may also change, which was altered to only a small extent in our studies. Previous study (Summerfield et al., 1985) showed that in lentils, ‘temperature had a much bigger effect on development (time to flowering) than photoperiod.’ Also, lentils of the Indian sub-continent are more sensitive to temperature and less sensitive to photoperiod, comparatively to genotypes of West Asia (Erskine et al., 1990).

Our findings indicated that heat stress was more detrimental than drought stress on biomass, seed number and seed yield in lentil while individual seed weight was influenced more by drought. Higher impact of heat stress than drought stress might be attributed to more duration of exposure of late-sown plants to stressful high temperatures involving reproductive and seed filling stages, while the exposure to drought stress was relatively lesser and involved only seed filling stage. Nevertheless, high temperatures in drought-stressed lentil plants at the time of seed filling severely influenced the biochemical processes associated with seed filling, causing a drastic reduction in seed quantity and quality. Consequently, seed yield was severely reduced, which was attributed to physiological and metabolic impairment of the photosynthetic components (Pn, chlorophyll fluorescence, chlorophyll concentration, stomatal conductance, sucrose concentration and starch metabolism) and water relations, which restricted the supply of sucrose to the developing seeds reducing their size and number. Genotypic variation was observed in response to drought and heat stress, whether alone or in combination, which was attributed to different metabolic responses in leaves and seeds.

### Leaf Function

Photoassimilates produced by leaves are used to maintain vegetative biomass as well as being transported to developing seeds. Photosynthetic efficiency in terms of carbon fixation and assimilation largely decides the rate and duration of seed filling. In this study, heat stress reduced vegetative biomass more than drought stress due to longer exposure to heat. Biomass reduction under stress may occur due to inhibition of growth-related metabolism (Rollins et al., 2013) involving numerous enzymes and hormones, which has been reported elsewhere (Barnabás et al., 2008).

Stress damages membranes; drought stress damaged the leaf tissue (measured as increased in electrolyte leakage) more than heat stress, and increased markedly further under the combined stress, possibly due to the direct impact of high temperature and increased water loss from leaf tissue. Electrolyte leakage from the stressed tissues indicates membrane instability due to changes in the lipid-protein configuration (Earnshaw, 1993) and leakage of vital ions and impaired cellular function (Conde et al., 2011), which intensifies under combined stresses. These observations match others reported in chickpea (Kumar et al., 2012) and Kentucky bluegrass (*Poa pratensis* L., Liu et al., 2008) exposed to heat stress and in chickpea (Awasthi et al., 2014) under combined drought and heat stress.

Plant growth and yield are strongly influenced by photosynthetic ability in the stress environment. Heat stress increased the Pn rate and RuBisCo activity, which was associated with an increase in *g*s resulting in more starch in leaves. These findings are similar to those found in chickpea leaves (Awasthi et al., 2014). Drought stress and the combined stress reduced Pn and RuBisCo activity, more so under the combined stress, which correlated with a reduction in *g*s thus inhibiting sucrose and starch concentrations in leaves and seeds. At the same time, chlorophyll concentration decreased under each stress treatment, likely due to the disruption of chloroplast membranes by direct or indirect effects such as photooxidation, which denatures the chlorophyll molecules (Kotak et al., 2007; Ristic et al., 2007; Djanaguiraman et al., 2010). Similar observations have been reported in bentgrass (*Agrostis stolonifera* L.) under combined heat and drought stress (McCann and Huang, 2007). The reduction in leaf chlorophyll concentration may have impaired Pn and RuBisCo activity in the stress environment. This can impact chlorophyll fluorescence (an indicator of PSII function and efficiency of the ‘light reaction’ of photosynthesis), which decreased more under drought stress than heat stress, and was further decreased with combined stress. The decrease in fluidity and electron dynamics due to stress may also inhibit PSII function (Havaux, 1992). The combined stress may degrade vital components of PSII, such as D1, D2 and CP47 proteins, to limit photosynthetic activity (Sainz et al., 2010). In this context, our observations are similar to those in *Lotus japonicus* (Regel) K. Larsen (Sainz et al., 2010) and chickpea (Awasthi et al., 2014).

RuBisCo, the carbon fixing enzyme, was inhibited under drought stress and the combined stress. Water deficit inhibits carbon fixation by closing stomata and reducing the flow of carbon dioxide into mesophyll tissue (Chaves et al., 2003), or by directly inhibiting the metabolic activity of enzymes (Farquhar et al., 1989). The decrease in RuBisCo activity reduced sucrose production in leaves and its availability to developing seeds, which affected seed weight and hence yield. RuBisCo activity under heat stress increased resulting in an increase in leaf starch concentration, but it decreased under drought, thus inhibiting starch accumulation. The combined stress severely inhibited sucrose and starch production, both in leaves and seeds, due to the exacerbation of individual effects of individual stresses on biosynthetic enzymes (Carmo-Silva et al., 2012). The flow of sucrose from leaves to seeds may be related to the down-regulation of sucrose transporters located in their tissues (Qin et al., 2008).

### Seed Function

Seed filling largely depends upon sucrose imported from the leaves as well as synthesized in the seeds (Yang et al., 2004). Impairments in photosynthesis as a result of heat and drought stress, imposed alone, or as combined treatment, reduced the production of sucrose and starch in the leaves as well as in the seeds. Metabolic events, associated with starch and sucrose metabolism, function in a coordinated manner, such that those occurring in developing seeds affect seed filling (Awasthi et al., 2014). The concentration of sucrose and starch varied between leaves and seeds, and under heat and drought stress, applied alone or in combination, which reflected their expression of metabolic enzymes. In general, starch and sucrose concentrations decreased markedly in developing seeds under the combined stress, which was attributed to severe inhibition of biosynthetic enzymes (sucrose-P-synthase, starch phosphorylase) as well as sucrose-utilizing enzymes (acid invertase). The reduction in sucrose concentration in leaves of drought-stressed lentil plants in our study match the observations in soybean (Liu et al., 2004), peach [*Prunus persica* (L.) Batsch.; Lo Bianco et al., 2003] and common bean (*Phaseolus vulgaris* L.; Castrillo, 1992). In an earlier study, the decrease in sucrose concentration was related to reduced enzyme activity related to sucrose synthesis such as sucrose–p-synthase or increased use of sucrose by the action of hydrolases such as invertase (Cornic and Massacci, 1996). Likewise, decrease in sucrose synthase activity has been reported in water-stressed bean plants (Castrillo, 1992). On the other hand, drought stress increased sucrose-synthesizing enzymes (sucrose synthase and sucrose phosphate synthase) in pigeon pea (Keller and Ludlow, 1993). Our observations of a severe reduction in sucrose under the combined stress are in contrast to findings in *Arabidopsis thaliana* (L.) Heynh. (Rizhsky et al., 2004) where sucrose concentrations were higher under combined heat and drought stress. These variations may be due to differences in species, plant age, or intensity and duration of the stress.

Sucrose imported by seeds is metabolized to glucose and fructose by various types of invertase isozymes such as vacuolar, cell-wall-bound and neutral invertases (Ruan, 2012). The activity of vacuolar acid invertase in lentil increased under heat and drought stress. The breakdown products are hexoses, which are used to power cellular processes, as building molecules for essential polymers such as starch and cellulose. The reducing sugars produced as a result of invertase action act in osmoregulation (Kameli and Losel, 1995). Similar to our study, drought stress in tomato (*Solanum lycopersicum* L.) leaves decreased sucrose synthase activity but increased invertase activity to reduce sucrose concentration (Bhatt et al., 2009). The combined stress markedly reduced the concentration of reducing sugars, which was related to the inhibition of β-amylase and acid invertase activity, suggesting a severe reduction in carbohydrate metabolism. The synthesis of sucrose and its utilization operate together to sustain photosynthetic processes and any inhibition in sucrose utilization would affect sucrose production (Nguyen-Quoc and Foyer, 2001) with negative consequences on the availability of precursors for starch accumulation.

Seed weight per plant as well as individual seed weights declined under drought and heat stress, especially the combined stress, possibly due to decrease in rate and duration of seed filling, resulting in fewer and smaller seeds, as reported earleir in soybean (Dornbos and Mullen, 1991) and chickpea (Awasthi et al., 2014). The decline in seed weight may be primarily ascribed to inhibition in starch accumulation, because of reduction in sucrose import to seeds and the inhibition of starch-synthesizing enzymes. Drought stress during grain development in cereals reduced the starch concentration, which was as a result of impaired capacity of the endosperm because of fewer amyloplasts (Jones et al., 1996). Earlier reports indicated adverse effects of environmental constraints such as drought and high temperature on seed development to affect seed quality (Triboï et al., 2000; Larmure et al., 2005). Similar to our findings, the activity of starch-synthesizing enzymes declined in wheat grains exposed to heat stress (Hawker and Jenner, 1993). Likewise, in wheat grains developing under water stress, a reduction in the accumulation of carbohydrates was observed, as a consequence of less availability of substrates for synthesis of starch and sucrose (Ahmadi and Baker, 2001). Our observations on severe reduction of seed yield in lentil due to combined stresses match those observed in chickpea (Awasthi et al., 2014) and wheat (Shah and Paulsen, 2003; Balla et al., 2011).

Similar to our observations on starch, the activity of starch-synthesizing enzymes in rice (*Oryza sativa* L.) declined subjected to drought and high-temperature environment, which reduced the starch concentration (Wang et al., 2006), thereby affecting seed size. The activity of β-amylase in our studies increased more under heat stress than drought stress, which was related to the increasing degradation of starch to produce more reducing sugars.

### Genotypes

We used genotypes contrasting in their sensitivity to drought and heat to determine their cross tolerance to drought and heat stress. The tolerant lentil genotypes (for heat and drought) performed significantly better than the sensitive genotypes, and produced more pods and seeds under individual or combined stress environments. Thus, our findings validate the categorization of these genotypes into tolerant or sensitive to heat or drought. We also noticed partial cross tolerance in HT and DT genotypes to the two stresses. One of the aims of having differentially-sensitive genotypes in our study was to probe the mechanisms related to tolerance to heat and drought stress. Our observations indicated that the tolerant genotypes could maintain higher chlorophyll and RLWC—which is associated with higher *g*s and photosynthetic function under stress environments—and may be attributed to better water extraction ability of the roots and *g*s. Consequently, these genotypes were able to sustain photosynthesis and carbon assimilation in leaves to support seed-filling processes.

Our findings are similar to some previous studies, where DT chickpea genotypes maintained higher RLWC (Deshmukh and Mate, 2013), PSII function (Mishra et al., 2012), *g*s (Yordanov et al., 2003) and membrane stability (Almeselmani et al., 2011). Similarly, under heat stress, tolerant lines in case of few other plant species showed less injury to leaves involving these traits (Srinivasan et al., 1996; Singh et al., 2007; Kumar et al., 2012). In the present study, tolerant lentil genotypes had significantly higher activities of RuBisCo, sucrose and starch-synthesizing enzymes than sensitive genotypes under all three stress environments, which may be due to the higher RLWC. Previous studies have shown that tolerant genotypes in case of other plant species can maintain high activity of RuBisCo (Ji et al., 2012), sucrose synthase (Saeedipour, 2011) and starch synthase (Sumesh et al., 2008) under both drought and heat stress.

### Stresses

While drought stress during seed filling caused more damage to water relations (RLWC, osmotic potential, transpiration rate and osmotic potential) and photosynthetic function (Pn, RuBisCo, chlorophyll, PSII function), heat stress caused more reduction in yield traits (pod number, seed number, seed weight/plant), which may be due to the longer heat exposure during reproductive and seed-filling stages. Consequently, heat stress markedly reduced aboveground biomass, which limited the number of flowers, pods and seeds to inhibit seed yield. Interestingly, individual seed weights declined more under drought stress than heat stress, which was attributed to the greater impact of drought stress on sucrose and starch production in seeds. The combined stress exacerbated the damage to water relations and photosynthesis, likely due to more reduction in water coupled with high temperature, which severely decreased yield traits in all the genotypes.

### Organs

Leaves and seeds varied in their sucrose and starch metabolism. In leaves, sucrose synthesis was promoted under heat stress, especially in tolerant genotypes, but not under drought stress or the combined stress, and matched the activity of the sucrose-synthesizing enzyme (sucrose-P-synthase). In contrast, sucrose concentration and SPS activity declined in seeds under all stress treatments, but less so under heat stress. Drought stress affected starch accumulation more than heat stress in both leaves and seeds. The reduction in seed weight due to stress is primarily attributed to a reduction in starch accumulation, which was linked to a reduction in the activity of the starch-synthesizing enzyme as well as poor availability of sucrose to seeds. Moreover, sucrose hydrolysis by acid invertase increased in leaves under drought and heat stress, but not under the combined stress. In seeds, sucrose hydrolysis increased under heat stress only. In contrast, starch hydrolysis by β-amylase increased under drought as well as heat stress in both leaves and seeds, with some exceptions in sensitive genotypes. The end products of both enzymes are reducing sugars, which increased in leaves and seeds, more so under drought stress than heat stress, unlike the expression of these enzymes, suggesting differences in the response of two organs. Nevertheless, the combined stress was highly detrimental to carbohydrate metabolism in both leaves and seeds, resulting in stunted plants, and fewer and smaller seeds.

Pod number, pod weight and seed number/plant were positively correlated with biomass and seed yield under the combined heat and drought stress treatment (**Table 6**). In contrast, membrane damage was negatively correlated with biomass and seed yield/plant. RLWC, osmotic potential and stomatal conductance had positive correlations with biomass and seed yield/plant, as did photosynthetic traits (Pn, RuBisCo, chlorophyll). Leaf and seed sucrose concentrations were more strongly correlated with seed yield than leaf and seed starch concentrations. Sucrose synthase in seeds was more strongly correlated with seed yield than starch phosphorylase. The activity of enzyme acid invertase and the concentration of reducing sugars in leaves and seeds were also strongly correlated with seed yield. The analysis indicated a strong, coordinated involvement of various traits in impacting seed yield suggesting that several components function together in deciding the plant’s response to combined stresses.

## Conclusion

In our study, heat stress reduced seed yield more than drought stress, while the combined stress severely inhibited seed size and weight. The combined stress also markedly impaired photosynthetic ability, water relations and carbohydrate metabolism in both leaves and seeds, resulting in stunted plants with fewer and smaller seeds. The reduction in seed size and weight was mainly attributed to a drastic reduction in sucrose and starch-synthesizing enzymes. The observations on contrasting lentil genotypes indicated partial cross tolerance to drought and heat stress in tolerant genotypes, which needs to be explored further with more genotypes.

## Author Contributions

The experimental work was done by AS and KS. The germplasm was provided by JK, SK, and SS. The data analysis and manuscript was written by first two authors and HN. The Ms was edited by KS.

## Conflict of Interest Statement

The authors declare that the research was conducted in the absence of any commercial or financial relationships that could be construed as a potential conflict of interest.
